# IL-4 induces the formation of multinucleated giant cells and expression of β5 integrin in central giant cell lesion

**DOI:** 10.4317/medoral.20935

**Published:** 2016-12-06

**Authors:** Amirala Aghbali, Sona Rafieyan, Leila Mohamed-Khosroshahi, Behzad Baradaran, Dariush Shanehbandi, Maryam Kouhsoltani

**Affiliations:** 1 Dental and Periodontal Research Center and Department of Oral and Maxillofacial Pathology, Faculty of Dentistry, Tabriz University of Medical Sciences, Tabriz, Iran; 2 Immunology Research Center, Tabriz University of Medical Sciences, Tabriz, Iran; 3 Immunology Research Center and Department of Oral and Maxillofacial Pathology, Faculty of Dentistry, Tabriz University of Medical Sciences, Tabriz, Iran

## Abstract

**Background:**

It is now well established that IL-4 has a central role in the development of monocytes to multinucleated giant cells (MGCs) by inducing the expression of integrins on the surface of monocytes. The aim of this study was to investigate the potential role of IL-4 in induction of β5 integrin expression in the peripheral blood samples of patients with giant cell granuloma.

**Material and Methods:**

Monocytes were isolated from peripheral blood samples of patients with central giant cell granuloma (CGCG) and healthy controls using human Monocyte Isolation Kit II. Isolated monocytes were then cultured in the absence or presence of IL-4 (10 and 20 ng/mL), and following RNA extraction and cDNA synthesis, Real-time PCR was performed to determine the level of β5 integrin expression. The formation of CGCGs and morphological analyses were done under light microscopy. For confirmation of CGCGs, immunocytochemistry technique was also carried out by anti-RANK (receptor-activator of NF-κB ligand) antibody.

**Results:**

In both patient and control groups, β5 levels were significantly enhanced by increasing the IL-4 dose from 10 to 20 ng/mL. In addition, these differences were significant between patient and control groups without IL-4 treatment. On the other hand, the number of cells which expressed RANK and therefore the number of giant cells were significantly higher in the patient group in comparison to controls, as assessed by immunohistochemistry evaluations.

**Conclusions:**

In this study, we showed an elevation in the expression levels of β5 integrin when stimulated by IL-4. It is strongly indicated that this integrin acts as an important mediator during macrophage to macrophage fusion and development of giant cells.

**Key words:**β5 integrin, giant cell, Il-4, monocyte, rank.

## Introduction

Central giant cell granuloma (CGCG) and peripheral giant cell granuloma (PGCG) are non-neoplastic lesions of jaw bones typically containing many MGCs (1). These lesions share common histopathological features and usually occur in the gums as well as alveolar mucosa of mandibular and maxillary bone (2,3). CGCGs are intraosseous non-neoplastic proliferative lesions and solely occur in maxillary bones (4). As to histology, this type of osteolytic lesions share some features with PGCG such as proliferation of fibrous tissue, hemosiderin deposits, hemorrhagic foci, reactive bone formation and osteoclast-like giant cells (5). CGCGs are more aggressive and typically tend to relapse. Unlike CGCG, PGCG is reactive, extraosseous and exophytic, settled in the alveolar ridge in the gingiva (6,7). Although these lesions were observed for the first time in 1953 by Jaffe (8), their exact origin ‎and clear etiology are not well elucidated despite huge amount of research. ‎In the last few years, macrophages, osteoclast precursors and some osteoblastic cells have been noted in the CGCG and PGCG derived mononuclear cells (9). Previously, some studies have revealed that probably the giant cells of these lesions are formed from the mononuclear cells (10). However, the exact association between giant cells and mononuclear cells has not been clearly understood (11).

It is proposed that inflammatory-immune mechanisms might be behind the pathogenesis of these lesions. This idea was confirmed by the presence of the osteoclast-like cells interacting with mononuclear cells (12). In individuals with CGCG, a change in the number of circulating lymphocytes and monocytes producing anti-inflammatory and inflammatory cytokines, including interleukin-3 (IL-3), IL-4, IL-10, and IL-13 is observed (10). IL-4 particularly, can act as a pivotal element in regulating the differentiation and functional activity of monocyte-macrophage lineage cells (13). There is now accumulating evidence that IL-4 can promote the formation of MGC in vitro through relatively unknown mechanisms (14). In one of these studies, it was shown that IL-4 prevents the differentiation of myeloid precursors to osteoclasts, which is mediated by the RANK through STAT6-dependent signaling pathway (15). Therefore, it was proposed that IL-4 might act as a molecular switch between osteoclasts and MGCs (16). In addition, IL-4 can regulate the expression of E-cadherin on the cell surface, causing homotypic cell fusion and the formation of giant cells (13).

The integrins, a superfamily of cell adhesion molecules, mediate cell-cell and cell-extracellular matrix (ECM) interactions. These heterodimeric molecules comprise of α and β subunits and are important meditators of signal transduction between extra- and intracellular environments (17). Some members of β1 and β2 integrins family are expressed by monocytes/macrophages. Therefore, it is suggested that these families are necessary and sufficient directors of adhesion in monocyte-to-macrophage development and IL-4-induced MGC formation (18).

In other words, IL-4 is supposed to cause an increase in the expression of this specific subgroup of integrins and also integrin receptors, leading to advancement of the mentioned process. In the present study, we aimed to investigate the potential induction of β5 expression in response to IL-4 (19). β5 is a novel β subunit and promotes cellular attachment to vitronectin and fibronectin ligands exclusively and is also involved in the morphological transformation of monocytes to giant cells.

## Material and Methods

- Patients and Ethics 

Blood samples were obtained from 5 CGCG patients and healthy donors were also used as control. All participants have given written informed consent and the study protocol was conducted in accordance with the Ethics Committee of Tabriz University of Medical Sciences which was in compliance with the Helsinki declaration.

- Monocytes Isolation 

Human blood-derived monocytes from patients with giant cell granuloma and controls were isolated from whole blood drawn from healthy individuals. Briefly, a 1:3 blood/PBS dilution was placed over Histopaque density gradient (Histopaq-1077, Sigma-Aldrich, St. Louis, MO), followed by centrifugation at 500 g for 25 min. The interface was separated and washed three times with PBS. Monocytes were isolated using the human Monocyte Isolation Kit II (Milteny Biotec, Auburn, CA). Approximately 50 million cells obtained from the Histopaque separation were resuspended in 500 µl of degassed buffer (PBS without  Ca++ and Mg++ at a pH of 7.2 + 2 mM EDTA + 0.5% BSA), followed by the addition of 100 µl of the BiotinAntibody Cocktail and 100 µl of FcR Blocking Reagent. The cell suspension was incubated at 4o C for 10 min. 250 µl of the buffer and 250 µl Anti-Biotin Microbeads were added to the cell suspension. After the cells were incubated for 50 min at 4o C, 5 ml of the buffer was added to the cell suspension, and the cell suspension was centrifuged at 250 g for 12 min. Cells were resuspended in 500 µl of the buffer and run through a Magnetic Separation LS column (Milteny Biotec). 10 ml of the buffer was added to the column, and the effluent 6 (monocytes) was collected. The monocyte suspension was centrifuged at 250 g for 15 min, and the cell pellet was resuspended in RPMI + 10% FCS. Monocyte purity was determined by flow cytometry based on the percentage of CD14 positive cells. The purity was greater than 90%.

- Cell Culture and Treatment

Isolated monocytes were cultured in 25cm2 flasks in RPMI 1640 medium (Invitrogen, Life Technologies, Carlsbad, CA) supplemented with 15% fetal bovine serum (FBS), 2 mM L-glutamine, 100 units/ml of penicillin-streptomycin and then were treated with two different doses (10 and 20 ng/mL) of IL-4 (R & D Systems, Minneapolis, MN) for 5 days. Results represent the mean ± SEM of data from three different monocyte donors.

- RNA extraction and real-time PCR analysis 

b5 integrin and mRNA levels were quantified by qRT-PCR. Total RNA was isolated from cells using RNeasy Mini Kit (Qiagen, Valencia, CA) according to the manufacturer’s instructions. Complementary DNA (cDNA) was synthesized by cDNA synthesis kit (Qiagen) from 1 μg of total RNA. Following on, qRT-PCR was performed in the Rotor-GeneTM 6000 system (Corbett Life Science, Australia). The PCR reaction conditions: 5 μL of cDNA template, 0.5 μM of each primer, 12 μL of SYBR green reagent and 7 μL of nuclease-free distilled water. The b5 integrin primer sequences were as follows: forward, 5’-GCC TAT CTC CAC GCA CAC TG-3’, reverse, 5’-AGA CTC CGA CCC TTC CTG AC-3’; and GAPDH, forward 5’-TCT AGA CGG CAG GTC AGG TCC ACC-3’ and reverse, 5’-CCA CCC ATG GCA AAT TCC ATG GCA-3’ were used as internal controls. Cycling conditions were as follows: 94°C for 5 min for cDNA and primer denaturing, followed by 35 cycles at 94°C for 30 sec, 56°C for 45 sec, and 72°C for 60 sec. Relative b5 integrin mRNA expression was determined with the 2 - (ΔΔCt) method using 18S as the reference gene.

- MGC formation analysis

Isolated monocytes from CGCGs plated in 24-well plates in the absence or presence of IL-4 (10 and 20 ng/mL). After 5 days, the plates were fixed and MGC formation was observed under light microscopy (Nikon, Japan). Also, immunocytochemical analysis by using a biotin-streptavidin-peroxidase staining method (LSAB2 kit, DAKO, Carpinteria, CA) was used to measure the relative quantities of RANK protein and MGCs formation. RANK protein expression on the surface of the monocytes is an evidence for monocyte to giant cell development. To determine RANK, we used rabbit anti-human RANK antibody at 1:2,000 dilution (Abcam, Cambridge, UK). Anti-RANK antibody was directed against the RANK’s extracellular domain. After Anti-RANK incubation, the cells were treated with 3% H2O2 in PBS for 10 min to inactivate endogenous peroxidase activity. Sequential incubations then were performed with biotinylated secondary antibody for 2 hrs. and peroxidase-conjugated streptavidin (DAKO). Staining was revealed with 3,3’-diaminobenzidine (DAB, Sigma Chemical Co., St. Louis, Mo.). The DAB areas in slides are brown (highlighted DAB-immunostained slides with the RANK antibody); the expression levels of RANK quantified by measuring the intensity of brown area by ImageJ software (National Institues of Health, Bethesda, Maryland, USA). Percentage for each sample represents the mean ± standard deviation (S.D.) of three independent experiments with 3 determinations.

- Statistical analysis 

All data were presented as mean±standard deviation (SD) of three independent experiments, and statistical analysis was performed using SPSS 16.0 software through t test method. Values of *P* less than 0.05 were considered statistically significant.

## Results

**Figure 1:**
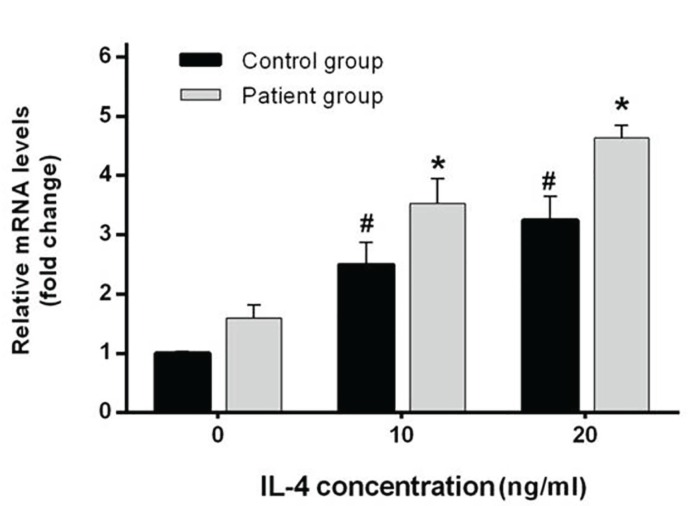
Up-regulation of β5 Integrin mRNA by IL-4. Monocytes were isolated from CGCGs and healthy donors were plated in 25cm2 flasks in the absence or presence of IL-4 (10 and 20 ng/mL). Media and IL-4 were replaced every other day. After 5 days, β5 integrin mRNA levels were examined by Real-time-PCR and relative mRNA expression levels were quantified by the 2 (-ΔΔCt) method. The results represent mean±SD (n=3); * and # *p*<0.05.

**Figure 2:**
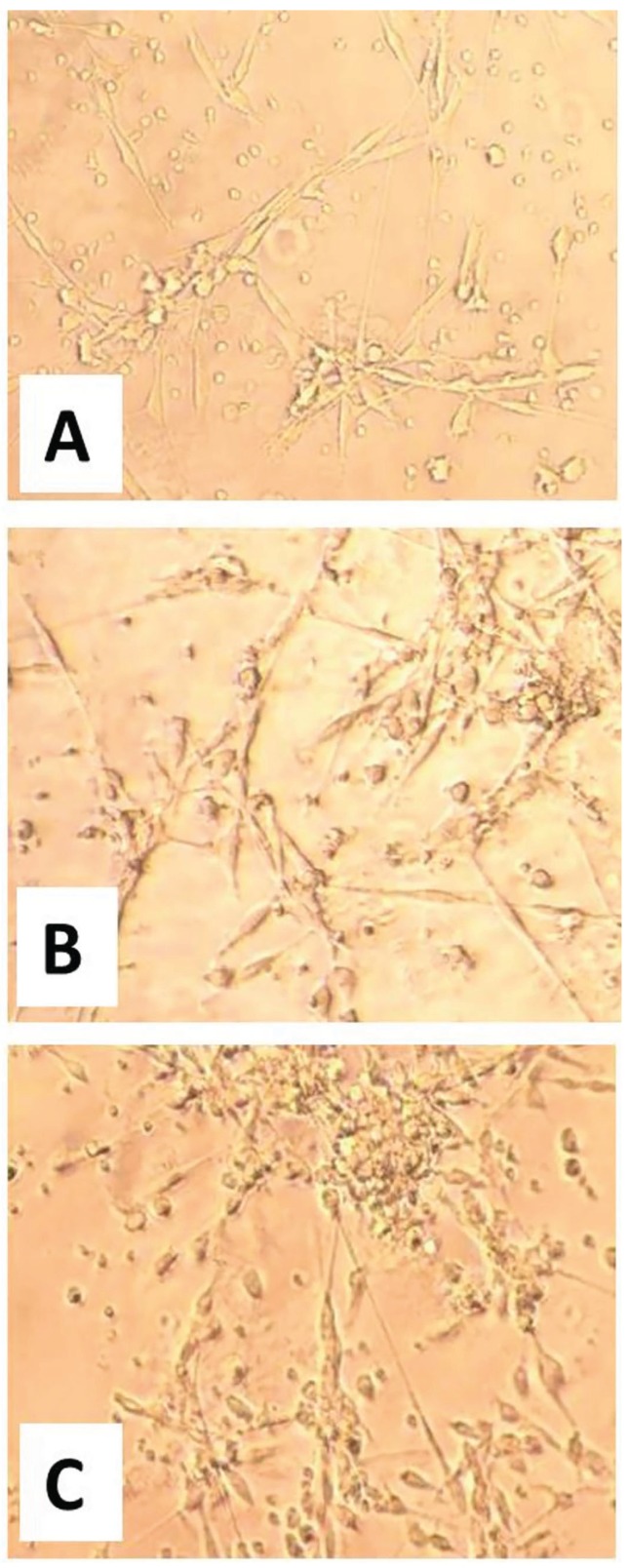
MGC formation is dependent on IL-4 concentration. Monocytes were isolated from CGCGs and plated in 24-well plates in the absence or presence of IL-4 (10 and 20 ng/mL). After 5 days, the cells were fixed and MGC morphology and formation was observed under light microscopy (40×). (A) Absence of IL-4; (B) Cells after IL-4 treatment (10 ng/mL), and (C) Cells after IL-4 treatment (20 ng/mL).

**Figure 3:**
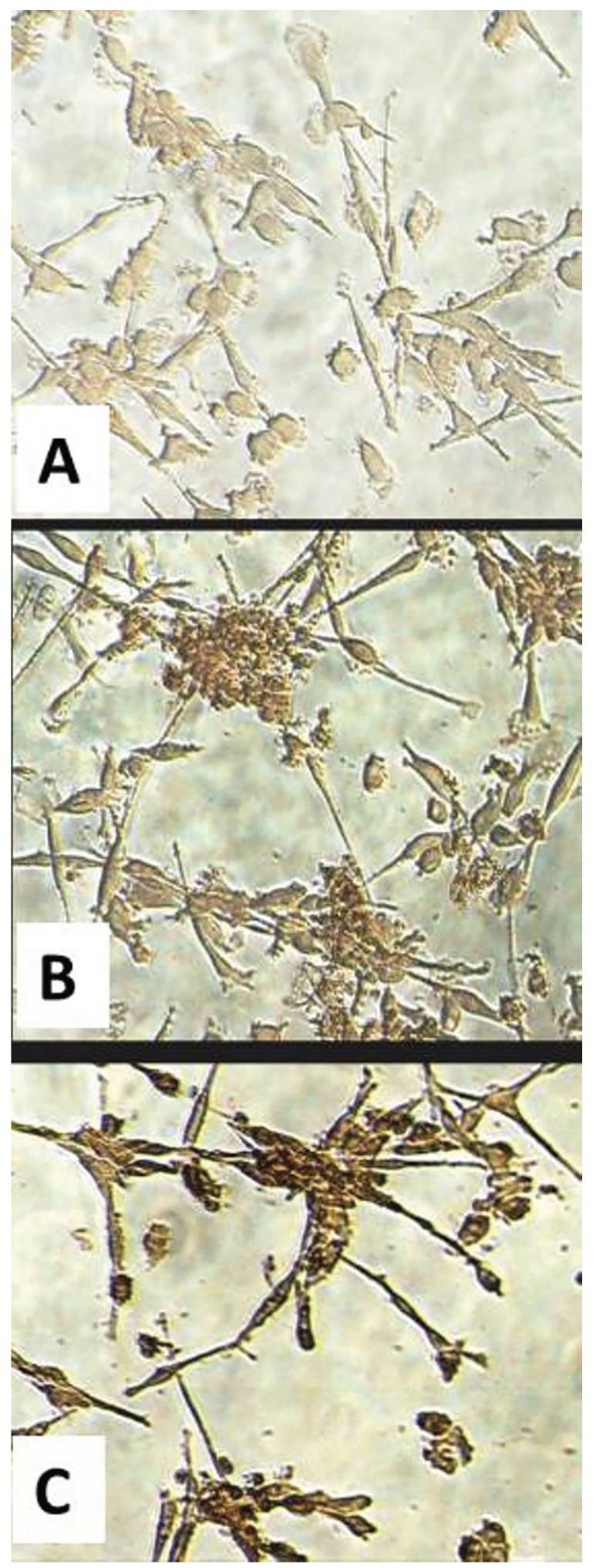
Inductive effect of IL-4 on RANK protein expression and MGC formation. Immunocytochemistry results using anti-RANK Abs in monocytes isolated from CGCGs and plated in 24-well plates in the absence or presence of IL-4 (10 and 20 ng/mL). After 5 days, the cells were stained with DAB. (A) Absence of IL-4; (B) Cells after IL-4 treatment (10 ng/mL), and (C) Cells after IL-4 treatment (20 ng/mL). The intensity of DAB staining (brown stain) shows the RANK protein expression levels.

Five days after IL-4 treatment, the expression level of β5 integrin was evaluated in CGCGs and healthy groups by Real-time PCR. The results showed that an increase in IL-4 dose from 10 to 20 ng/mL causes a statistically significant elevation in expression levels of β5 integrin in patient and control samples (*p*=0.01 and *p*=0.02, respectively). In addition, the differences between expression levels of β5 integrin, when compared between patient and control group, were significant bghv (*p*= 0.03 in the absence of IL-4, *p*<0.001 for samples treated with 10 ng/mL IL-4, and p=0.001 for samples treated with 20 ng/mL IL-4). In other words, the expression levels in different treated doses were higher in patients group in comparison with healthy controls. More interestingly, these differences were significant between patient and control groups without IL-4 treatment (Fig. 1). Examination of cultured cells by light microscopy in CGCGs group showed that treatment with IL-4 causes an alternation in monocyte morphology toward MGC formation (Fig. 2). For evaluating the effects of IL-4 on induction of RANK expression and MGC formation, we used immunocytochemistry by anti-RANK antibody (Fig. 3). RANK protein is overexpressed by neoplastic giant cells, promoting fusion with macrophage colony stimulating factor (M-CSF) acting as a cofactor. In immunocytochemistry analyses of patient samples, the numbers of the giant cells formed by IL-4 induction in 10 ng/mL and 20 ng/mL IL-4 concentration were significantly increased as compared with those of samples without IL-4 (*p*=0.01 and *p*=0.00, respectively). In patient samples, in the absence of IL-4 the percentage of giant cells was 31%, however in samples treated with IL-4, 73% of cells treated with 10 ng/mL IL-4 and 90% of cells treated with 20 ng/mL IL-4 were giant cells (*p*=0.01, *p*=0.03). These amounts are lower in control samples, 61% and 77% in 10 and 20 ng/mL of IL-4, respectively (Fig. 3).

## Discussion

To the best of our knowledge, this is the first study showing the involvement of b5 integrin in the monocyte to giant cell transformation and pathological identity of giant cell granuloma. IL-4 treatment increases the expression of the b5 integrin on the monocyte surface and induces ‎morphological changes toward MGCs in a dose-dependent manner.

From the biological point of view, the most attractive and significant feature of giant cells is that they are actually monocyte-derived multinucleated macrophages, formed by macrophage to macrophages fusion. However, the precise molecular mechanism of macrophage fusion has not yet been elucidated. In this regard, a very hot research front is the well-known and interesting role of IL-4 in the formation of giant cells. As a result, there have been several studies which investigated and discussed this important issue. For instance, Kondo and co-workers (20), studied the effects of different monoclonal cytokines (IL-4, granulocyte-macrophage colony-stimulating factor (GM-CSF), macrophage colony-stimulating factor (M-CSF), Interferon gamma (IFN-g), Tumor necrosis factor α (TNF-α) on a population of monocytes from cord blood and adult peripheral blood to investigate if they can induce MGCs. They reported that among these cytokines, IL-4 alone as well as IL-4 in combination with M-CSF and/or GM-CSF could increase MGC formation in both adult peripheral blood and cord blood culture, significantly. In another study, an alternative mechanism was proposed for IL-4 mediated induction of MGC formation. In this study, Yu and *et al.* (21) showed that NF-κB signaling is also required for IL-4-induced MGC formation. Additionally, IL-4 lead to over-expression of p105/p50 and stimulated the nuclear accumulation of p50. Furthermore, it was shown that the over-expression of NF-κB dimer proteins enhances the macrophage fusion; while on the other hand, p100 knockdown enhanced macrophage fusion. These results indicate that NF-κB pathway controls macrophage fusion induced by IL-4 treatment. Furthermore, Moreno and *et al.* (13) investigated the mechanism by which IL-4 promotes MGC formation, identifying a critical role for E-cadherin in the formation of giant cells, likely by modulating homotypic fusion of precursors. They found that the IL-4-induced production of MGCs is STAT6-dependent, based on the fact that precursors derived from STAT6-/- mice could not develop efficiently into giant cells upon treatment with IL-4. In agreement with these findings, in our study, treatment with IL-4 induced the formation of the MGCs in a dose-dependent manner from monocytes derived from patients with giant cell granuloma.

During the last decade, a number of studies have been performed to elucidate the exact molecular mechanism involved in homotypic macrophage-macrophage fusion. Based on the results of these studies, it has been suggested that specific groups of proteins mediate macrophage-macrophage fusion. Among these protein groups, integrin receptor families, particularly β1 and β2 have been both recognized as sufficient and essential mediators of adhesion during monocyte-to-macrophage alternation and IL-4-induced MGC formation (22). Complement family proteins and fibrinogen are known to be early adhesion ligands to the β2 integrins and, later, vitronectin has been recognized as the pivotal protein adhesion substrate for IL-4-induced MGC formation (18). McNally and *et al.* (17) designed a study to explore the underlying cell/substrate interactions which support optimal IL-4-induced macrophage fusion and MGC formation. They were also evaluating the potential involvement of integrin in these adhesion events. They demonstrated that β2 integrins are required for initial monocyte adhesion; however, during IL-4-induced macrophage fusion and MGC formation, both β1 and β2 integrins are necessary to mediate adhesion. Therefore, β1 and β2 integrins cooperate in cell adhesion during the formation of MGCs. On the other hand, another study revealed that fusing macrophages and foreign body giant cells (FBGCs) express an explicit group of adhesion receptors, namely aX and aM of the β2 integrin, and a2, a3, a5, and aV of the β1 integrin subfamily. This expression pattern indicated that the acquisition of fusion capabilities by macrophages during the formation of giant cells happens in parallel with the selective expression of specific adhesion molecules which might have the potential to interact with blood and ECM proteins (23,24). Therefore, since integrins play such important roles in the MGC formation, we investigated the expression levels of β5 integrin after treatment with IL-4. Similar to other integrins, β5 integrin was over-expressed on the surface of the monocytes upon IL-4 treatment.

In conclusion, IL-4 induced elevation of β5 integrin expression, as shown by real-time PCR and immunocytochemistry, indicates that β5 integrin can play a key role in mediating the macrophage to macrophage fusion and development of the giant cells in central giant cell granuloma. Therefore, this study and further future investigations will help to decipher of clear mechanisms underlying MGC formation. Subsequently, this will help us to come up with possible therapeutic targets for treatment of the central giant cell granuloma. One limitation of the study was that due to the ethical issues the least required amount of blood was collected from the subjects, therefore the experiments were not repeated several times to achieve more accurate mean values.
